# Superficial Peroneal Nerve Motor Branch Transfer to the Deep Peroneal Nerve: Cadaveric Study and Case Report

**DOI:** 10.1097/GOX.0000000000006781

**Published:** 2025-05-09

**Authors:** Adam J. Mosa, Zachary D. Randall, Brendan J. Navarro, Daniel A. Hunter, David M. Brogan, Christopher J. Dy

**Affiliations:** From the Department of Orthopaedic Surgery, Washington University in St. Louis School of Medicine, St. Louis, MO.

## Abstract

**Background:**

Foot drop carries substantial morbidity and is often due to deep peroneal (DPN) or common peroneal nerve (CPN) injury. Treatment options are limited. This study explored a new surgical approach by transferring a superficial peroneal nerve (SPN) branch to DPN. Cadaveric analysis, manual histomorphometry, and a case report are presented.

**Methods:**

Twenty-one limbs were analyzed. A reproducible surgical approach was used to identify CPN and trace it to the bifurcation into SPN and DPN, and then to the insertions into the peroneus longus (PL) and tibialis anterior (TA) muscles, respectively. Measurements were made from the superior most aspect of the fibular head to the bifurcation of the CPN, the insertion of the first and second SPN motor branches to the PL, and to the insertion of the DPN into the TA. The first SPN motor branch to the PL and DPN into the TA nerves were harvested, and histomorphological measurements of axonal densities were obtained.

**Results:**

Histomorphological analysis showed similar axonal densities between the transferred and target nerves, indicating a comparable potential for effective reinnervation. The mean distances from fibular head to various nerve branches were recorded to ensure tension-free transfer. No significant differences were found between nerve groups regarding axon density, total fascicle area, or total axon counts.

**Conclusions:**

This study supported feasibility of this nerve transfer technique, with initial results suggesting it represents a viable treatment option for foot drop secondary to DPN injury. Further research is needed to confirm these findings.

**Takeaways**

**Question:** Is there a potential to improve outcomes for patients with foot drop caused by deep peroneal nerve (DPN) injury utilizing a nerve transfer from the superficial peroneal nerve?

**Findings:** Utilizing cadaveric analysis, manual histomorphometry, and a representative case report, this study supported the feasibility of a superficial peroneal nerve to DPN nerve transfer technique, with initial results suggesting it represents a viable treatment option for foot drop secondary to DPN injury.

**Meaning:** In addition to more widely performed tendon transfer techniques and nonoperative orthotics, this study supported further exploration of a nerve transfer to restore active dorsiflexion in patients with foot drop.

## INTRODUCTION

Peroneal nerve (PN) dysfunction from trauma, iatrogenic injury, or compressive masses dramatically alters quality of life by increasing the risk of falls and associated morbidity.^1^ PN injuries can occur anywhere along the neurological axis but frequently result from lesions to the deep peroneal nerve (DPN) or common peroneal nerve (CPN).^2,3^ Treatment options for peroneal neuropathy are dictated by the etiology, which can include traumatic, oncological, musculoskeletal, spinal, and autoimmune disorders.^4^ Management options range from nonsurgical treatments such as physical therapy, orthotics, and electric stimulation, to surgical interventions typically focusing on nerve decompression or tendon transfers.^2,5–9^ Although the use of nerve transfers in treating isolated neuropathies has been shown to be successful in numerous anatomic locations,^10–12^ foot drop has less commonly been treated with nerve transfer.^11^ Recent studies, however, have reported the use of nerve transfers to the tibialis anterior (TA) motor branch for the treatment of foot drop. The most common donor is a motor branch or collection of motor branches of the tibial nerve. However, although some centers report good outcomes using these techniques,^13,14^ studies from other institutions have been unable to replicate these results.^15,16^

The anatomic feasibility of a peroneus longus (PL) motor branch of the superficial peroneal nerve (SPN) to TA nerve transfer was proposed in 1999.^17^ In the past 25 years, there have been no published studies further investigating the clinical utility of this proposed transfer or replication of the initial study describing technical feasibility based on nerve length and diameter. Contemporary nerve transfer literature frequently benefits from an objective analysis of axonal density in donor and recipient targets to ensure appropriate axonal density in the regenerating nerve. Such an axonometric analysis has not been performed for the PL to TA nerve transfer. There is consensus on the prerequisites for a successful nerve transfer. First, the “donor distal, recipient proximal” dictum, that is, the functioning donor nerve, has adequate length to reach the distal aspect of the recipient nerve without tension. Ideally, axonal parity or a close approximation is achieved to improve the chance of reinnervation.^18,19^ Finally, the gain of function to the recipient muscle must outweigh the loss of function to the muscle supplied by the donor nerve.^20,21^

Here we revisit the role and indications for a nerve transfer involving a donor PL branch of the SPN to a denervated TA by targeting the TA branch of the DPN. A demonstrative case example is provided, followed by a cadaveric analysis of landmarks and measurements to assist this transfer. Finally, axonometric analysis of recipient and donor nerves is provided. This transfer may be considered in the armamentarium for peripheral nerve surgeons treating patients with foot drop from isolated DPN injury.

## METHODS

Due to the nature of this study as a single case example in combination with a cadaver study, which does not constitute human subjects research, further human subjects research review was not applicable in accordance with our institution’s policies.

## CASE EXAMPLE

A 45-year-old woman with no preexisting medical history was referred to our complex nerve clinic with a clinical foot drop. One month prior, she was pinned between 2 vehicles and sustained an isolated traumatic right bicondylar tibial plateau fracture with immediate loss of dorsiflexion. She presented to a nearby hospital where she was initially stabilized with external fixation, followed by open reduction and internal fixation using a medial and lateral extensile approach. According to operative reports, her trauma surgeon identified an intact but contused common PN with surrounding edema.

At the time of consultation, she had persistent peroneal neuropathy with loss of sensation in the dorsal foot, absent ankle dorsiflexion, and no toe extension. Ankle eversion and PL activity were graded as MRC 3 out of 5. Her pain was well controlled without the need for narcotic analgesia. She was referred to our physiatry colleagues for assessment with ultrasound and electrodiagnostic studies. Sonographically, the anterior and lateral muscle compartments revealed increased echogenicity, suggesting muscle denervation, and the common PN was diffusely enlarged with a maximal cross-sectional area of 27 mm^2^. Nerve conduction studies revealed no response of the right peroneal motor nerve to the extensor digitorum brevis or TA. Electromyography of the TA and PL showed increased insertional activity and a markedly decreased interference pattern. The remainder of the electrodiagnostic studies were normal.

After these investigations, the surgical plan was exploration, neurolysis, and repair of the PN with an anticipated interposition nerve autograft. Intraoperatively, the PN was exposed and decompressed in a standard fashion.^22^ A neuroma in continuity was identified at the DPN, and no stimulation with a handheld Doppler was found in the deep peroneal branches. Branches of the SPN were identified proximal to the zone of injury, and these responded well to a handheld nerve stimulator at 0.5 mA. Two branches to the PL were identified, and both were stimulated with resultant ankle eversion. Given the redundancy of eversion with each branch to the PL, we elected to proceed with the nerve transfer to provide axons closer to the target for the TA and avoid the downsides of 2-nerve coaptation. Based on prior intraoperative findings, this nerve transfer was being considered, and the patient was informed preoperatively of the potential to perform a nerve transfer if the anatomy was favorable, that is, redundancy existed in the PL. One branch to the PL was then transected, and internal neurolysis was performed proximally to gain length. This was then transferred in an end-to-end fashion to the TA branch of the DPN, and a single-stand sural nerve autograft was used to reconstruct the DPN, which sent distal branches to the toe extensors. All coaptations were performed with a 9-0 nylon suture using a floor-mounted microscope. Finally, because the branching pattern of the SPN was exposed and dissected as part of the initial approach, the addition of the nerve transfer did not significantly impact operative timing, taking approximately 15 minutes extra.

Early postoperative recovery was uneventful and involved regular therapy visits for motor retraining and range of motion. At 4 months, the patient had a positive tender muscle sign^23^ over the lateral and anterior compartments and demonstrated MRC 2 for lesser toe dorsiflexion and ankle dorsiflexion with MRC 4 out of 5 for ankle eversion. At 12 months, she had MRC 4 for lesser toe dorsiflexion and ankle dorsiflexion and was ambulating without an orthosis.

**Fig. 1.**
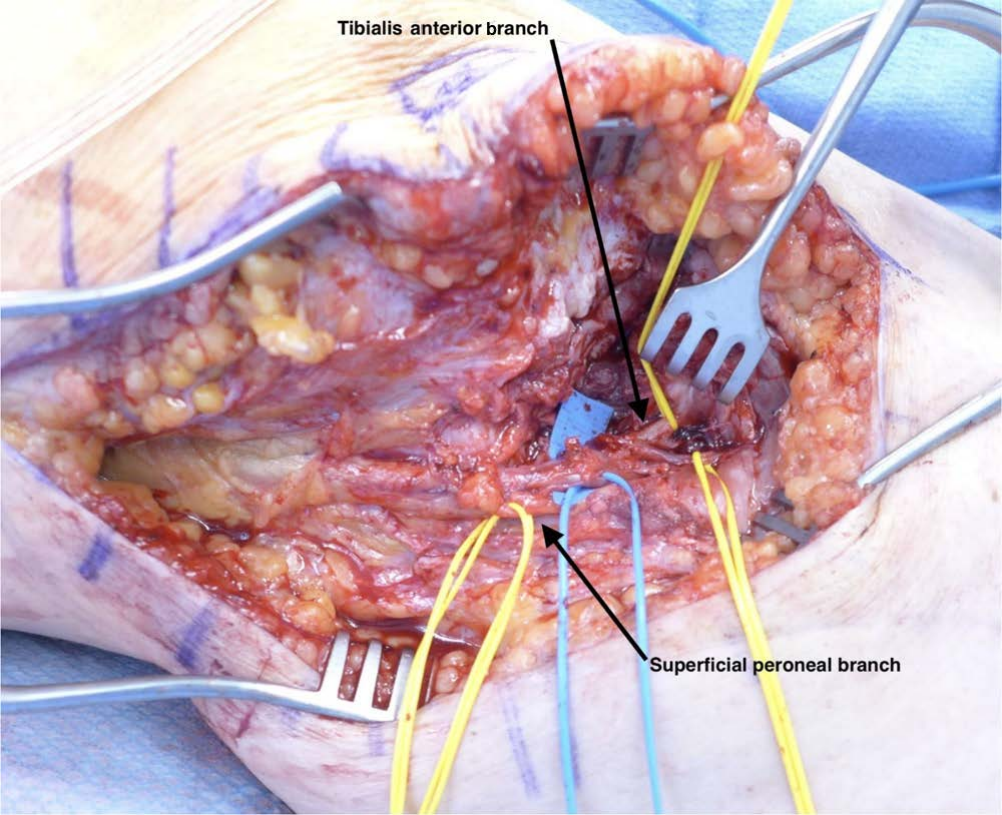
Preparation and identification of donors and recipients in the clinical case.

Twelve fresh-frozen cadavers were available for dissection, yielding 21 usable limbs for investigation. Limbs deemed unsuitable for dissection were excluded due to factors such as the presence of adjacent hardware, traumatic injury to the dissection area, or compromised tissue quality. Each dissection started with palpation to locate the fibular head and common PN. A curvilinear incision was made inferior to the fibular head, extending from posterior-proximal to anterior-distal along the common PN’s trajectory. To enhance exposure, the anterior-distal incision was extended inferiorly (Fig. 1).

Fascial incisions to expose the CPN and distal branches were performed from the popliteal fossa to the innominate intermuscular septum after first identifying and releasing the posterior and anterior crural septa. The longitudinal extension of the fascial release to permit visualization of nerve branches was performed in line with the posterior septum. The common PN was traced to the bifurcation into the superficial nerve and DPN. The superficial nerve was dissected until reaching the insertion points of its first and second branches into the peroneal longus muscle. Similarly, the DPN was followed by the motor branch’s insertion into the TA muscle.

Measurements were taken from the proximal aspect of the fibular head to 4 specified locations: the common peroneal bifurcation, the insertion points of the first and second motor branches of the SPN into the peroneal longus muscle, and the DPN branch insertion into the TA. All measurements were documented in centimeters.

**Fig. 2.**
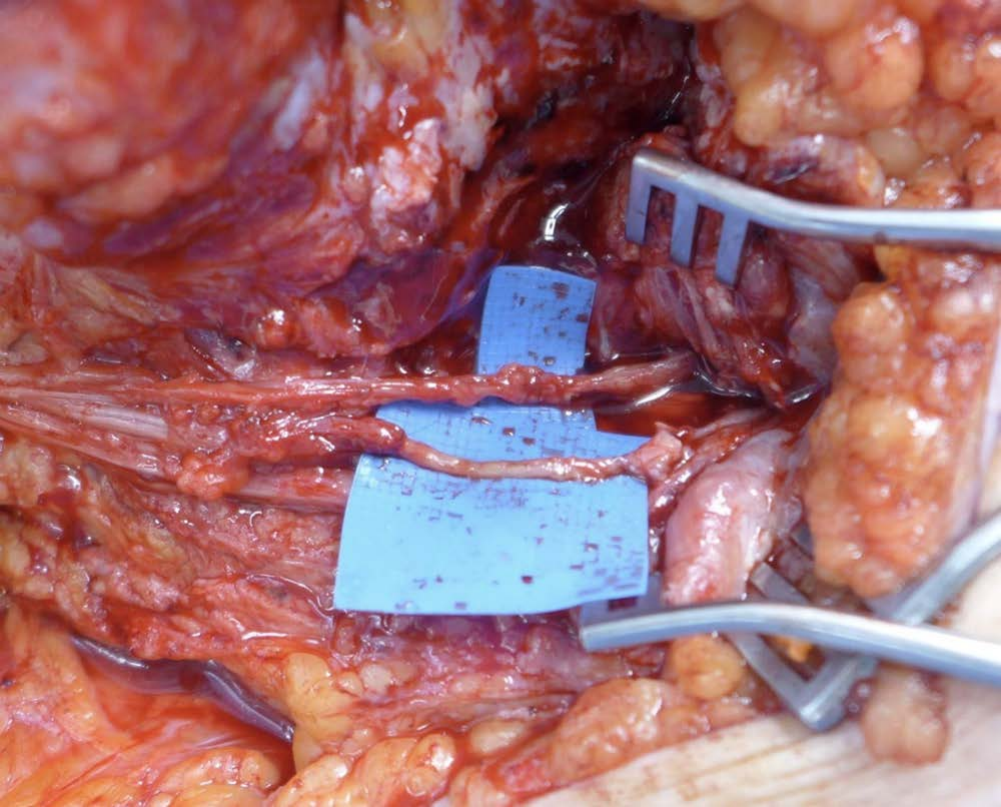
Clinical photograph after in situ transfer and interposition nerve graft.

Following measurement, nerve branches corresponding to the SPN’s first branch to the peroneal longus muscle (SPN-PL1, Fig. 2) and the DPN branch to the TA muscle (DPN-TA) were harvested for axon density analysis. These nerve specimens were mounted on wooden sticks and preserved in capped test tubes containing 3% glutaraldehyde in sodium phosphate buffer.

Collected nerve grafts were then trimmed into appropriate samples. The samples were fixed in 1% osmium tetroxide and serially dehydrated using various concentrations of ethanol. Once dehydrated the samples were embedded in Araldite 502 epoxy resin and cross-sectioned using an ultramicrotome (LKB Bromma 8800 Ultrotome III, LKB Instruments, Mount Waverly, Victoria) in 1.5 µm sections. These sections were then counterstained with 1% toluidine blue and imaged for analyses at 1000× overall magnification on a Leitz Laborlux S microscope (Leica Microsystems, Wetzlar, Germany).

For analysis, a semiautomated digital image analysis system linked to morphometry macros developed for peripheral nerve analysis (Clemex Vision Professional, Clemex Technologies, Longueuil, Quebec, Canada) was used.^24^ Six to 8 random fields per histological section were imaged, resulting in 75%–80% of the cross-sectional area being analyzed. Manual histomorphometry measurements were obtained for the total fascicular area and the total number of myelinated axons in the relevant nerve sections. The quantification of myelinated axons across multiple randomly selected fields per nerve permitted the calculation of the total number of myelinated axons, the area covered by myelinated axons (µm^2^), and the density of myelinated axons (per mm^2^).

### Statistical Analysis

Descriptive statistics consisted of means with SDs for continuous variables. All statistical analyses were performed using R (version 4.3.3, R Foundation for Statistical Computing, Vienna, Austria).

## RESULTS

**Fig. 3.**
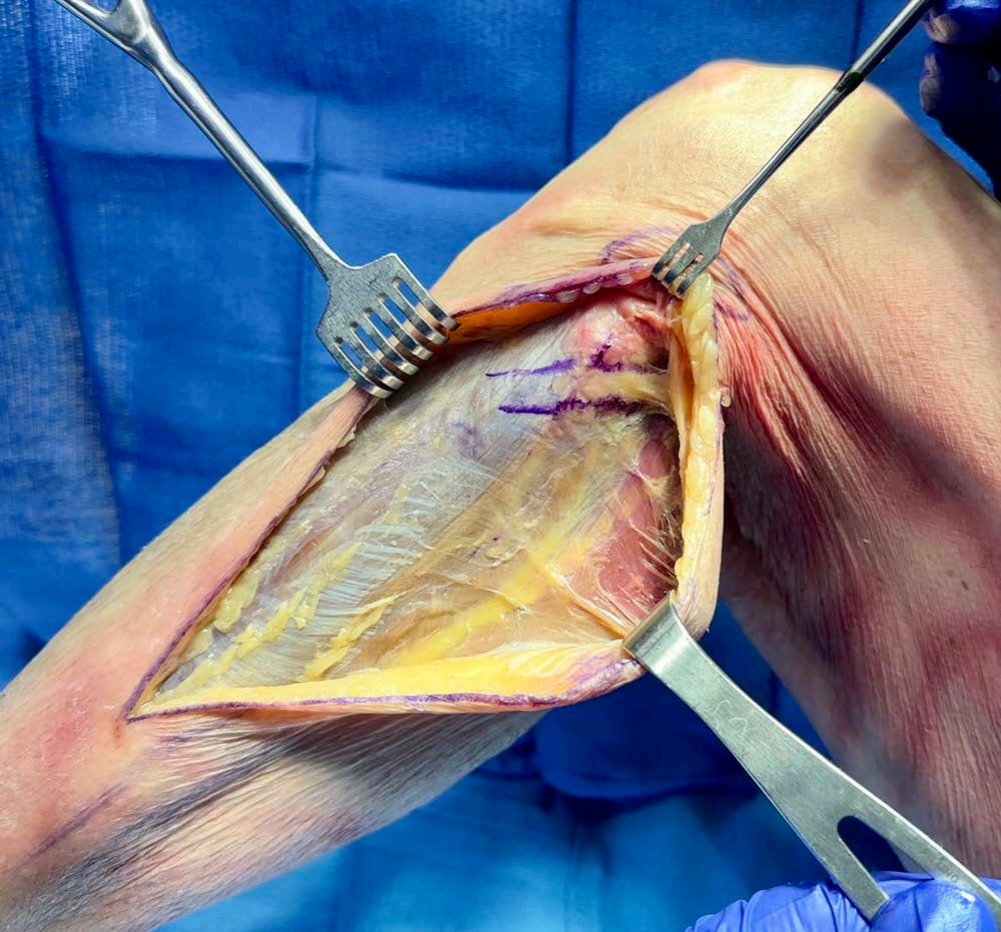
Subcutaneous dissection. Marker indicates the course of the palpable PN distal to the fibular head.

**Fig. 4.**
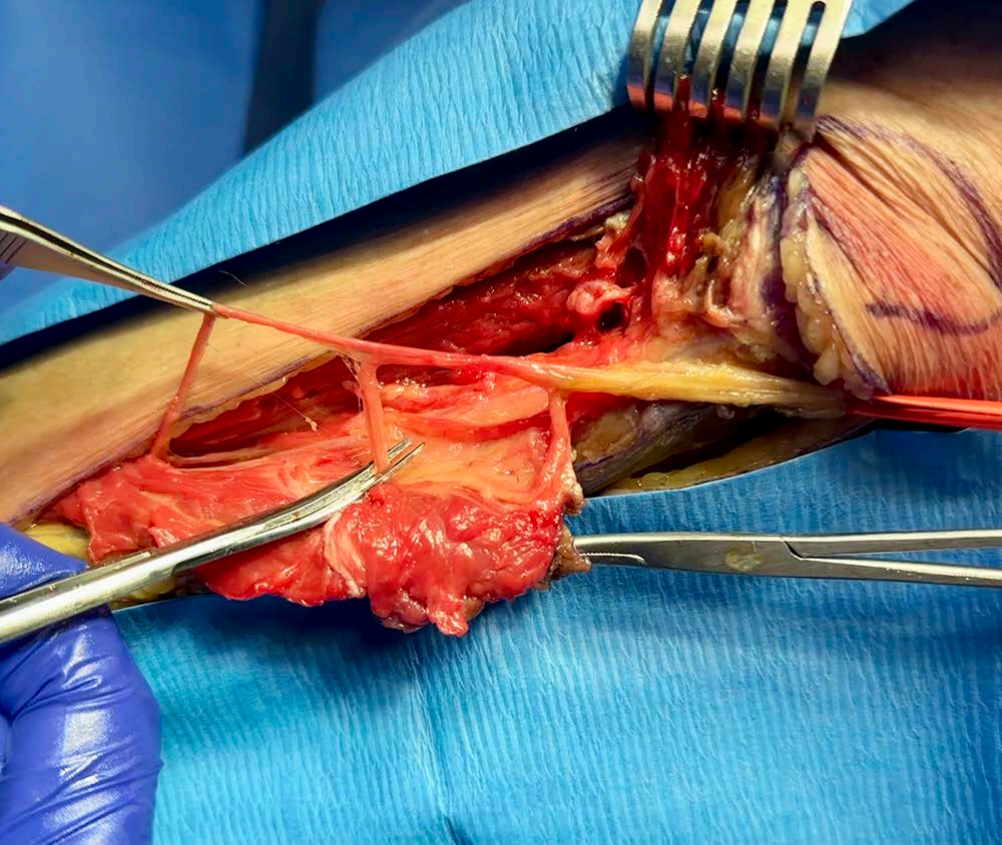
Harvesting the SPN branch to the PL muscle. The nerve is held under traction for demonstration purposes. Scissors are positioned at the location of the transection before the nerve becomes intramuscular.

**Fig. 5.**
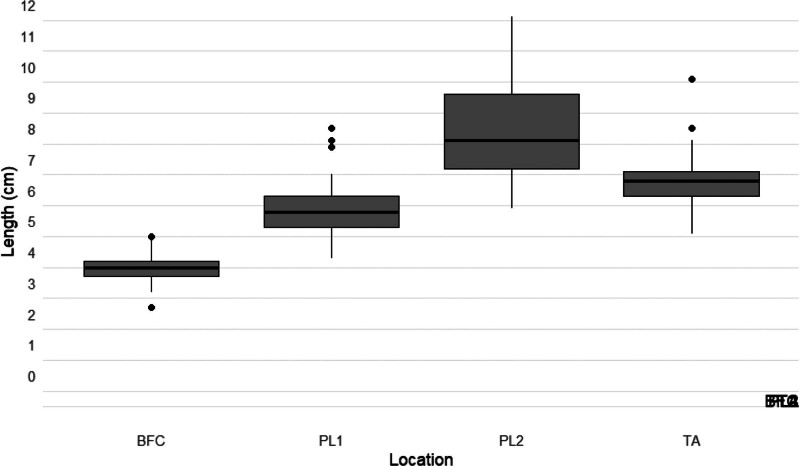
PN branching distances. BFC, bifurcation; PL1, 1st branch to peroneus longus; PL2, 2nd branch to peroneus longus; TA, branch tibialis anterior

Twelve adult fresh-frozen cadavers, comprising 8 female and 4 male specimens, provided a total of 21 dissectible limbs. Individualized measurements for each cadaver are detailed in Table 1, with distances for 21 CPN bifurcations, 21 SPN-PL1, 17 SPN’s second branch to the peroneal longus muscle, and DPN-TA. The mean CPN bifurcation distance was found to be 3.48 cm (SD = 0.57) from the proximal aspect of the fibular head, with individual measurements ranging from 2.20 to 4.50 cm. For SPN-PL1, the mean length measured 5.45 cm (SD = 1.17), ranging from 3.80 to 8.00 cm. Additionally, measurements for SPN’s second branch to the peroneal longus muscle showed a mean length of 8.04 cm (SD = 1.80), with individual values ranging from 5.40 to 11.60 cm. The DPN-TA had a mean length of 6.40 cm (SD = 1.07), ranging from 4.60 to 9.60 cm (Table 1, Figures 3–5). The provided measurements from the fixed landmark of the fibular head were chosen to improve intraoperative reproducibility.

**Table 1.** Anatomic Results

Individual	Side	Sex	CPN Bifurcation (cm)—Distance From Proximal Aspect of Fibular Head	SPN-PL1, cm	SPN-PL2, cm	DPN-TA, cm
1	Right	Male	3.2	5.1	—	6.4
1	Left	Male	2.8	8	—	8
2	Right	Female	3.4	5.3	—	7.6
2	Left	Female	2.2	7.6	—	5.6
3	Right	Female	3.5	5.2	11.6	5.6
3	Left	Female	3.2	5.6	9.8	6.8
4	Right	Female	4.4	5.3	8.9	6.2
4	Left	Female	2.8	7.4	11.5	6.3
5	Right	Male	4.5	3.8	6.7	9.6
5	Left	Male	3.3	5	8.5	4.8
7	Right	Female	3.7	4.3	6.4	4.6
7	Left	Female	2.7	5.8	9.1	6.4
8	Left	Female	3.3	4.2	5.4	5.4
9	Right	Female	3.4	5.1	6.3	6.3
10	Right	Female	3.7	5.4	7.6	6.6
10	Left	Female	3.9	6.2	7.4	5.8
11	Right	Male	3.6	6.5	7.8	6.7
11	Left	Male	3.6	4.3	6.5	6.4
12	Right	Female	4.4	4.8	9.4	6.3
12	Left	Female	3.6	3.8	6.7	6.3
13	Left	Male	3.8	5.8	7.1	6.6
Mean	—	—	3.48	5.45	8.04	6.40
Min	—	—	2.20	3.80	5.40	4.60
Max	—	—	4.50	8	11.60	9.60
SD	—	—	0.57	1.17	1.80	1.07

Measured from superior most aspect of the fibular head to muscular insertion of each specific nerve.

SPN-PL2, second peroneal longus branch of the SPN.

### Laboratory Results

**Fig. 6.**
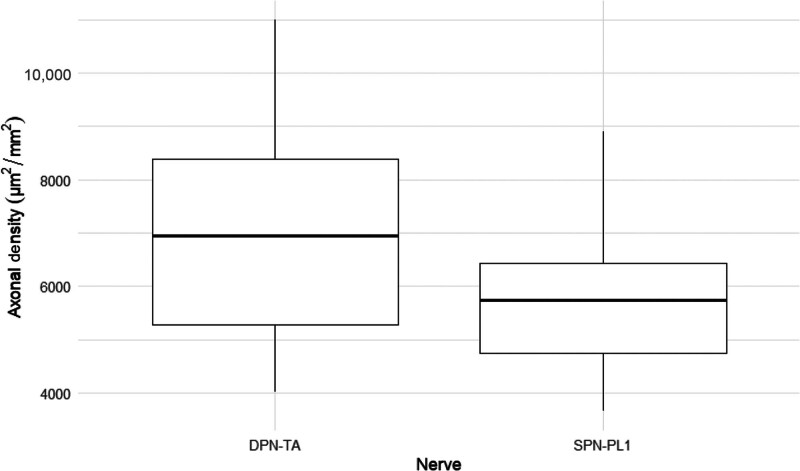
Axonal density measurements.

Descriptive statistics for the DPN-TA nerve showed a mean axon density of 7241.7 µm^2^/mm^2^ (SD = 2337, IQR = 5285–8380), a mean total fascicle area of 171,900 mm^2^ (SD = 146,366, IQR = 87,733–202,641), and mean total axons of 1438.3 (SD = 1744, IQR = 574–1551). For the SPN-PL1 nerve, the mean axon density was 5781 µm^2^/mm^2^ (SD = 1355, IQR = 4737–6423), the mean total fascicle area was 88,786 mm^2^ (SD = 45,705, IQR = 58,350–114,817), and the mean total axons were 503 (SD = 258, IQR = 319–585). The Mann-Whitney *t* test indicated no statistically significant differences between the 2 nerve groups in axon density (*P* = 0.11). There were statistically significant differences in total fascicle area (*P* = 0.02) and total axons (*P* = 0.01). The average donor-to-recipient axonal count ratio was 34.9% (Table 2, Fig. 6).

**Table 2.** Axonometric Analysis

	DPN-TA	SPN-PL1	*t* Statistic	*P*
Axonal density, mean, µm^2^/mm^2^	7242	5781	1.98	0.062
Fascicle area, mean, mm^2^	171,900	88,786	1.97	0.069
Total axons, mean	1438	503	1.92	0.079

## DISCUSSION

In recent years, nerve transfer has emerged as a promising approach for treating nerve injuries. Although traditionally used in the upper extremity, its application in patients with foot drop is not well defined. The concept of transferring the PL motor branch of the SPN to the denervated TA muscle via the DPN branch presents a novel and potentially effective intervention. This approach aims to restore dorsiflexion and improve gait mechanics in patients with foot drop, thereby enhancing their functional outcomes and quality of life. The presented case study illustrates the clinical application and outcomes of the novel nerve transfer procedure. A 45-year-old woman with traumatic foot drop secondary to a bicondylar tibial plateau fracture underwent exploration, neurolysis, and nerve transfer. Despite dense peroneal neuropathy, the patient showed an early return of TA activation at 4 months. Motor retraining and range of motion therapy facilitated functional improvement, highlighting the potential efficacy of the procedure in restoring dorsiflexion and enhancing patient mobility. It should be explicitly stated that in the case presented and during the cadaveric dissection, there was more than 1 superficial peroneal branch to the lateral compartment and PL muscle. By utilizing 1 branch for the transfer and leaving 1 intact, eversion was preserved. A potential complication of this transfer would be compromising a peroneal branch when there is no redundancy of PL innervation, thereby weakening or eliminating eversion. An intraoperative nerve stimulation device made this possible. Without redundancy, we would proceed with the direct nerve graft repair in isolation.

This cadaveric analysis corroborates earlier work by Buyukmumcu et al^17^ in the 1999 study which originally explored this nerve transfer. Our study provided updated descriptions with landmarks to aid surgical planning. Measurements of relevant motor branch lengths and their relationships to target muscles aid in determining the feasibility and optimal approach for nerve transfer procedures. This work demonstrated that sufficient length can be achieved from the donor nerve to facilitate tension-free coaptation. Preliminary clinical results are promising and supported based on axonometric analysis. The average donor-to-recipient axonal count ratio measured at 34.9% meets the criteria elucidated by earlier studies on the minimal density to achieve functional muscle reinnervation.^25^ Larger prospective studies can help validate the efficacy of the described SPN motor branch transfer to the DPN-TA branch. Long-term follow-up data on functional outcomes, patient satisfaction, and complications are required, especially in comparison to the existing dominant treatment algorithms involving tendon transfer.

This study has several limitations. First, only a single case example is provided based on the novelty of this treatment approach in our clinical practice. This restricts the generalizability of findings to a broader patient population. Although the presented case focuses on a traumatic crush mechanism, theoretically, the proposed transfer would be suitable for any mechanism where the SPN branch to the PL has been spared and the target muscle remains susceptible to reinnervation.

## CONCLUSIONS

This study highlighted the potential of a novel nerve transfer approach for the treatment of foot drop secondary to DPN injury. The cadaveric analysis supports that an appropriate working length is available to support a tension-free coaptation, and the axonometric analysis supports the compatibility of the donor nerve. The initial case report provides promising early results, but continued research is warranted to establish long-term efficacy.

## DISCLOSURES

Drs. Dy and Brogan report the following potential conflict of interest: research funding and speaker fees from Checkpoint Surgical. The other authors have no financial interest to declare in relation to the content of this article.
